# HAG regimen improves survival in adult patients with hypocellular acute myeloid leukemia

**DOI:** 10.18632/oncotarget.6211

**Published:** 2015-10-21

**Authors:** Xiaoxia Hu, Weijun Fu, Libing Wang, Lei Gao, Shuqin Lü, Hao Xi, Huiying Qiu, Li Chen, Jie Chen, Xiong Ni, Xiaoqian Xu, Weiping Zhang, Jianmin Yang, Jianmin Wang, Xianmin Song

**Affiliations:** ^1^ Department of Hematology, Institute of Hematology, Changhai Hospital, Second Military Medical University, Shanghai, China; ^2^ Department of Hematology, Changzheng Hospital, Second Military Medical University, Shanghai, China

**Keywords:** hypocellular, acute myeloid leukemia, induction regimen, prognosis

## Abstract

**Background:**

Hypocellular acute myeloid leukemia (Hypo-AML) is a rare disease entity. Studies investigating the biological characteristics of hypo-AML have been largely lacking. We examined the clinical and biological characteristics, as well as treatment outcomes of hypo-AML in our institutes over a seven years period.

**Design and Methods:**

We retrospectively analyzed data on 631 adult AML patients diagnosed according to the French-American-British (FAB) classification and WHO classification of tumors of haematopoietic and lymphoid tissue, including 43 patients with hypo-AML. Biological variables, treatment outcomes and follow-up data on hypo-AML patients were analyzed.

**Results:**

Out of 631 AML patients, 47 (7.4%) were diagnosed as hypo-AML, out of which 43 patients were evaluable. Compared with non-hypocellular AML, hypo-AML patients tended to be older (*P* = 0.05), more likely to present with leukocytopenia (*P* < 0.01) and anterior hematological diseases (*P* = 0.02). The overall complete remission (CR) rate, disease free survival (DFS), and overall survival (OS) in hypo-AML patients were comparable to those in non-hypo AML patients. Twenty-seven (62.8%) patients with hypocellular AML were treated with the standard regimen of anthracyclines and cytarabine (XA) (associated CR rate: 51.9%; median OS: 7 months; median DFS: 6.5 months). Sixteen (37.2%) patients were treated with a priming regimen containing homoharringtonine, cytarabine and G-CSF (HAG) (associated CR rate: 81.25%; median OS: 16 months; median DFS: 16 months).

**Conclusions:**

The overall prognosis of hypo-AML was not inferior to that of non-hypo AML. HAG regimen might increase response rates and improve survival in hypo-AML patients.

## INTRODUCTION

While most cases of acute myeloid leukemia (AML) are hypercellular or normocellular, up to 5-12% of all cases are that of hypocellular AML (Hypo-AML).[1-5] The clinical course, diagnosis and management of nine cases of hypoplastic acute myelogeneous leukemia was first described in 1975.[3] In 1996, Nagai K et al proposed the diagnostic criteria for hypo-AML (cellularity of bone marrow < 40% and blast percentage > 30% in absolute nucleated cells (ANC).[6] Currently, hypo-AML is defined as bone marrow cellularity < 20% on biopsy, although in some earlier reports, cellularity less than 40% to 50% has been considered as indicating hypo-AML.[2, 4, 7, 8]

The treatment options for hypo-AML [9, 10], complete remission (CR) rates, overall survival (OS), duration of remission, and event-free survival (EFS) were found to be comparable to those of patients with non-hypocellular AML in a large study on 123 patients of hypo-AML and 1219 non-hypocellular AML. In this study, incidence of early death during the first four weeks was relatively high, which markedly influenced the OS. [9] So the optimal induction chemotherapy regimen remains undefined.

Because of its low incidence, the documented clinical experience with this type of adult leukemia is limited. We undertook this study to assess the frequency of hypo-AML among a large Chinese cohort of patients with AML. We describe the clinical features of this group and compare their clinical outcomes with those of other patients with AML, to assess the impact of this pathologic feature on prognosis, and to evaluate the efficacy of different induction regimens.

## RESULTS

### Clinical and biological characteristics

There were 631 patients with de novo AML seen at the department of Hematology, Changhai and Changzheng hospital during the study period. 47 patients (7.4%) were diagnosed as hypo-AML (bone marrow cellularity < 20%) based on bone marrow biopsy. One patient did not opt for therapy due to poor general condition. The other 3 patients were excluded due to lack of availability of complete data. 43 patients were evaluable for the study. The clinical features of patients with hypo-AML are summarized in Table 1. Of the remaining 584 non-hypocellular AML patients, 541 patients were evaluable.

**Table 1:** Patient characteristics by study group

	Hypo-AML	Non-hypo-AML	*P*
Total patients (N)	43	541	
Female sex, N (%)	21 (48.8)	229 (42.3)	0.42
Median age, y (range)	56 (20-65)	46 (15-78)	0.05
AHD, No. (%)	10 (23.2)	43 (8)	0.02
Median WBC count, ×10^9^/L (range)	2.2 (0.2-64.4)	12.6 (0.5-247)	< 0.01
Median HGB level, g/L (range)	83 (30-142)	82 (35-134)	0.8
Median platelet count, ×10^9^/L (range)	51 (4-306)	35 (1-374)	0.42
Median bone marrow cellularity, %(range)	14 (11-20)	56 (24-82)	0.03
Median blasts in bone marrow (%)	43 (24-85)	53 (20-96)	0.53
Cytogenetics, No. (%) Poor Intermediate Favorable	19 (44.2) 19 (44.2) 5 (11.6)	209 (38.6%) 275 (50.8%) 57 (10.5%)	0.72 0.81 0.24
CR, No. (%)	27 (62.8%)	303 (56.0%)	0.48
Median OS, months	9	8	0.7
Median DFS, months	8	10	0.9

Hypo-AML, Hypocellular acute myeloid leukemia; AHD, anterior hematological disease; HGB, hemoglobin; CR, complete remission; OS, overall survival; DFS, disease free survival

In this study, 22 males and 21 females were diagnosed with hypo-AML. Median age was 56 years (range: 20-65 years) and the median bone marrow cellularity was 14% (range: 11-20%). According to the French-American-British (FAB) classification, M2 (14/43, 32.5%), M5 (22/43, 51.2%), and M1 (7/43, 16.3%) were the most frequent subtypes (Table 2). The median white blood cell (WBC) count, hemoglobin (HB), platelets (PLT) were 2.2 (range: 0.2-64.4) ×10^9^/L, 83 (range: 30-142) g/L, and 51 (range: 4-306) ×10^9^/L, respectively. Four patients had a WBC count greater than 10.0×10^9^/L, and three of them had prior myeloproliferative neoplasm (MPN) but with evidence of fibrosis on biopsy. The median percentage of blasts in bone marrow was 43% (range: 24-85%). Ten patients had anterior hematological disease (AHD, 4 patients with MPN, and 6 patients with myelodysplastic syndrome [MDS]).

**Table 2:** Patient's distribution by FAB classification and mutation analysis

	N	%	Total
**FAB classification**			43
**M1**	7	16.3	
**M2**	14	32.5	
**M5**	22	51.2	
**Mutation analysis**			Analyzed (N, % of total) (37, 86%)
**NPM1**	3	8.1	
**FLT3-ITD**	2	5.4	

FAB, French-American-British; NPM1, nucleophosmin; FLT3-ITD, FMS-Like Receptor Tyrosine Kinase-3 Internal Tandem Duplication

In 21 patients, valid chromosome profiles were obtained during the therapy. The cytopreserved cells of the remaining 22 patients without cytogenetic information at diagnosis were retrieved and analyzed retrospectively. Five patients had t (8; 21) (q22; q22) or inv (16) (p13.1q22) or t (16; 16) (p13.1; q22). Six patients had complex chromosomal abnormalities. Nine patients had abnormalities in chromosome 7, two patients had −5q, and two patients had mixed lineage leukemia (MLL) rearrangement.

The nucleophosmin (NPM1) and FMS-Like Receptor Tyrosine Kinase-3 Internal Tandem Duplication (FLT3-ITD) mutation analysis was done in 37 (37/43, 86%) patients; only 2 were found positive for FLT3-ITD, and 3 were positive for NPM1 mutation (Table 2).

**Figure 1:**
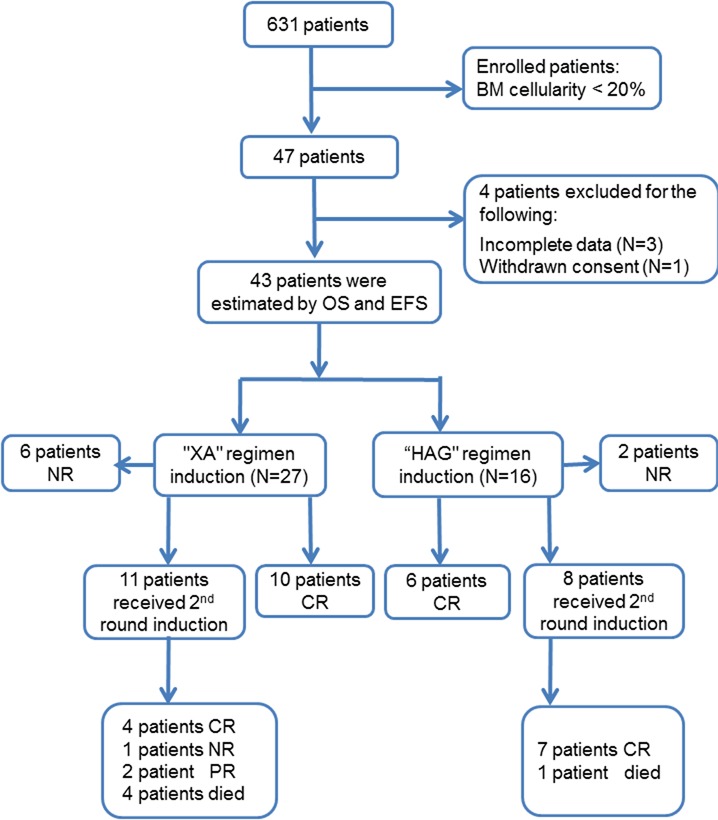
Study design and outcomes

### Clinical and biological characteristics of hypo- and non-hypo AML

**Figure 2:**
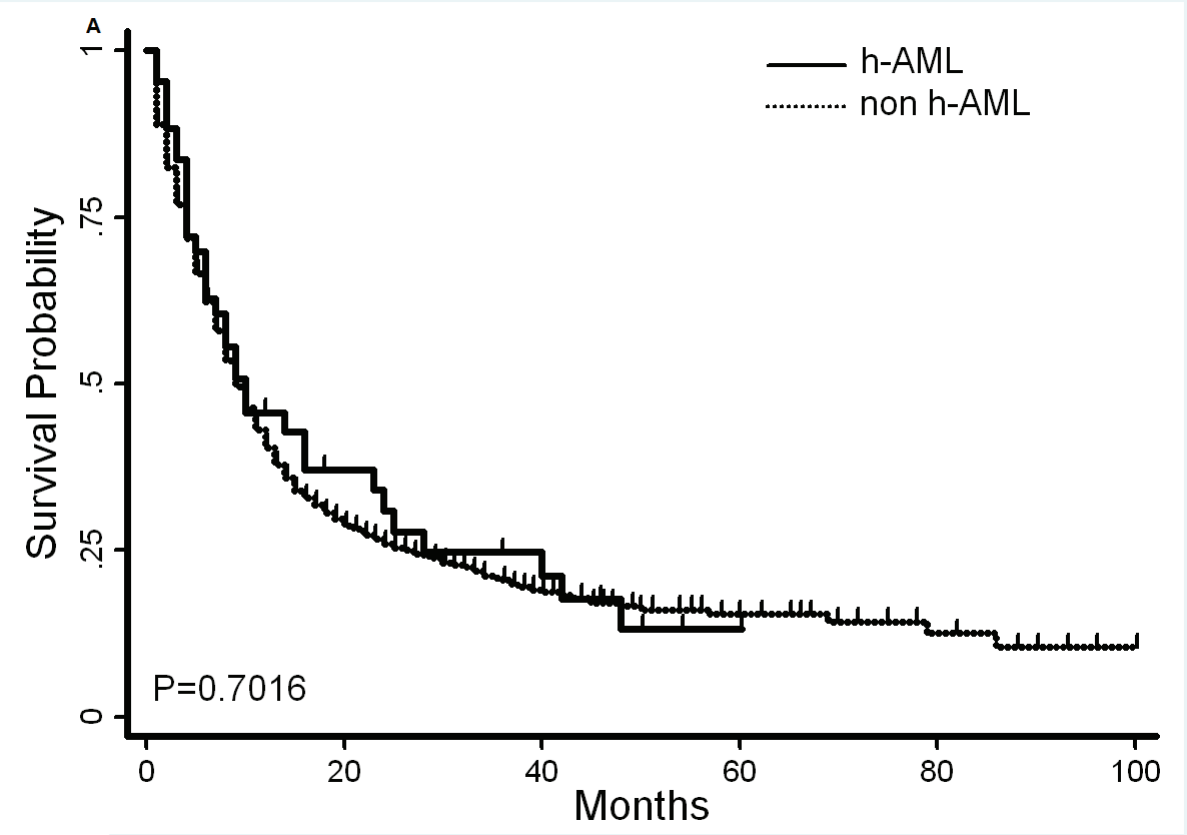

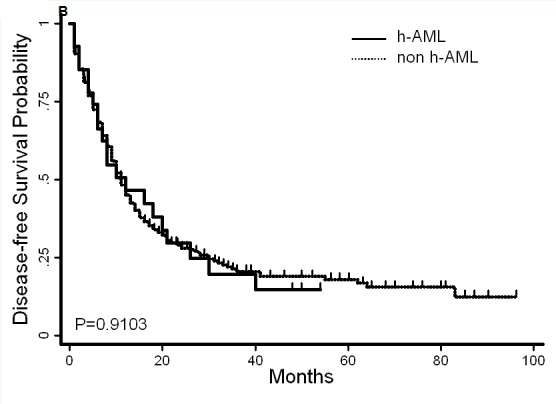
Overall survival and disease-free survival of AML patients with or without hypocellular feature The median survival time in patients with hypo-AML and non-hypo AML was 9 and 8 months, respectively **A.**, *P* = 0.7. The median disease-free survival was 8 and 10 months, respectively **B.,** *P* = 0.9.

Several differences were observed in the clinical presentation of patients with and without hypocellular AML. Hypo-AML was more frequently associated with AHD (*P* = 0.02), lower white blood cell counts (*P* < 0.01), and older age (*P* = 0.05). Other clinical characteristics such as hemoglobin (HB), platelet counts (PLT) and cytogenetic characteristics were comparable between the two groups. The CR rate for hypo-AML and non-hypo AML was 62.8% and 56%, respectively (*P* = 0.48). Median OS time was 9 months and 8 months, respectively. (*P* = 0.7, Figure 2A) Among patients who achieved CR, the median duration of disease-free survival (DFS) was 8 months and 10 months, respectively (*P* = 0.9, Figure 2B).

### Induction therapy and its predictive factors

The results of induction therapy are shown in Tables 3 and 4. A total of 16 out of 43 patients (37.2%) were prescribed a priming regimen containing homoharringtonine, cytarabine and G-CSF (HAG regimen), while 27 of 43 patients (62.8%) enrolled in the standard regimen cohort containing anthracyclines and cytarabine (“XA” regimen). Six patients (6/27, 22.2%) aged > 60 years received XA induction, while 5 patients (5/16, 31.25%) were treated with HAG (*P* = 0.719). In this study, frail patients and those with AHD were found more likely to have received HAG induction. Eight patients (8/43, 18.6%) did not achieve any responses with one cycle of induction (6 in XA group and 2 in HAG group). Nineteen patients who achieved partial remission (PR) with one cycle of induction were re-induced with the same induction regimen, out of which 11 (11/19, 57.9%) achieved CR. After 2 cycles of induction, 4 patients were found to have resistant disease. Fourteen patients achieved CR with XA regimen (14/27, 51.8%) and 13 patients with HAG (13/16, 81.25%). 1 patient (6.25%) died during induction in the HAG cohort as compared to 4 (14.8%) in the XA cohort (*P* = 0.635). No significant differences were observed between the causes of death during induction among the 2 induction regimens. During induction, patients induced with HAG regimen experienced a mildly prolonged myelosuppression. The duration of agranulocytosis was 7.8 days and 9.4 days in XA and HAG groups (*P* = 0.042). Duration of time for which the platelet count was < 20×10^9^/L was slightly longer in the HAG group as compared to that in the XA group (*P* = 0.061).

**Table 3:** Characteristics and clinical outcomes in hypo-AML patients induced with XA or HAG regimen

	XA	HAG	*P*
No. of patients	27	16	
Female sex, N (%)	13 (48.1)	8 (50)	1
Median age, y (range)	50 (29-65)	56.5 (20-65)	0.555
Age ≥ 60y (%)	6 (22.2)	5 (31.25)	0.719
AHD, N (%)	7 (25.9)	3 (18.75)	0.719
Performance status > 2	2 (7.4)	6 (37.5)	0.037
Cytogenetics, N (%) poor intermediate favorable	12 (44.4) 13 (48.4) 2 (7.4)	7 (43.75) 6 (37.5) 3 (18.75)	0.504
Duration of agranulocytosis (d)	7.8	9.4	0.042
Duration of platelets < 20 ×10^9^/L (d)	8.2	11.2	0.061
CR (%)	14(51.9)	13 (81.25)	0.101
Allo-HSCT	5 (18.5)	4 (25)	0.706
Median OS, months	7	16	0.0206
Median DFS, months	6.5	16	0.2316
Treatment related mortality	4 (14.8)	1 (6.25)	0.635

Hypo-AML, Hypocellular acute myeloid leukemia; XA, anthracyclines and cytarabine; HAG, homoharringtonine, cytarabine and G-CSF; AHD, anterior hematological disease; CR, complete remission; OS, overall survival; DFS, disease free survival.

**Table 4:** Characteristics of patients with CR versus no CR

Characteristics	CR	No CR	*P*
No. of patients	27	16	
Female sex, N (%)	15 (55.6)	6 (37.5)	0.256
Median age, y (range)	54 (20-65)	57.5 (35-65)	0.061
AHD, N (%)	3 (11.11)	7 (43.75)	0.021
Median WBC count, ×10^9^/L (range)	1.6 (0.3-51.6)	3.2 (1.2-13.3)	0.472
Median HGB level, g/L (range)	89 (40-151)	76.5 (48-109)	0.211
Median platelet count, ×10^9^/L (range)	53 (9-212)	50.5 (6-248)	0.323
Cytogenetics, N (%) poor intermediate favorable	7 (25.9) 15 (55.6) 5 (18.5)	12 (75) 4 (25) 0	0.003
Induction treatment, N (%) XA regimen HAG regimen	14 (51.9) 13 (48.1)	13 (81.25) 3 (18.75)	0.062

CR, complete remission; AHD, anterior hematological disease; HGB, hemoglobin; XA, anthracyclines and cytarabine; HAG, homoharringtonine, cytarabine and G-CSF;

The characteristics of patients with hypo-AML with or without CR are shown in Table 4. On univariate analysis, AHD and poor cytogenetics appeared to be associated with induction failure. XA induction regimen was marginally associated with treatment failure (*P* = 0.062). On multivariate analysis, poor cytogenetics and induction with XA regimen were independent predictive factors for induction failure (Table 5).

**Table 5:** Multivariate analysis for covariates for complete remission

Characteristic	Odds ratio	*P*
Age	0.9088 (0.8225, 1.0042)	0.060
AHD	5.4849 (0.8002, 37.5967)	0.083
Cytogenetics	0.0684 (0.0090, 0.5208)	0.010
Induction treatment regimen	16.6404 (1.4128, 195.9899)	0.025

AHD, anterior hematological disease

### Clinical outcomes

**Figure 3:**
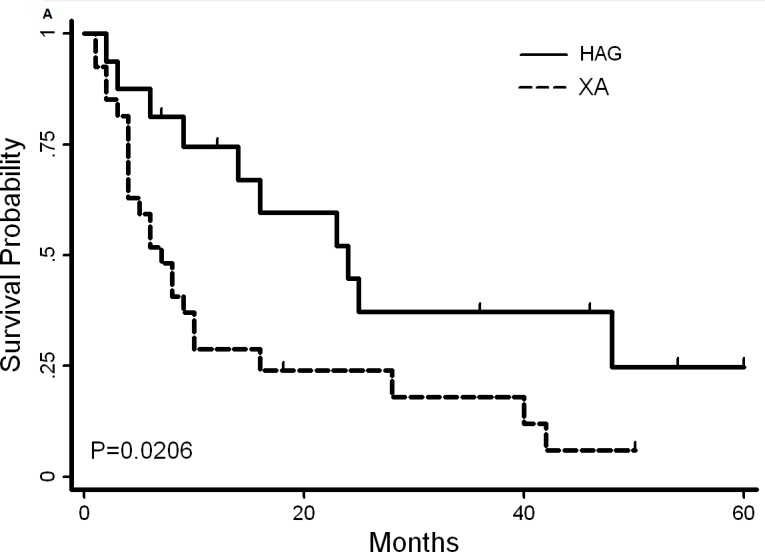

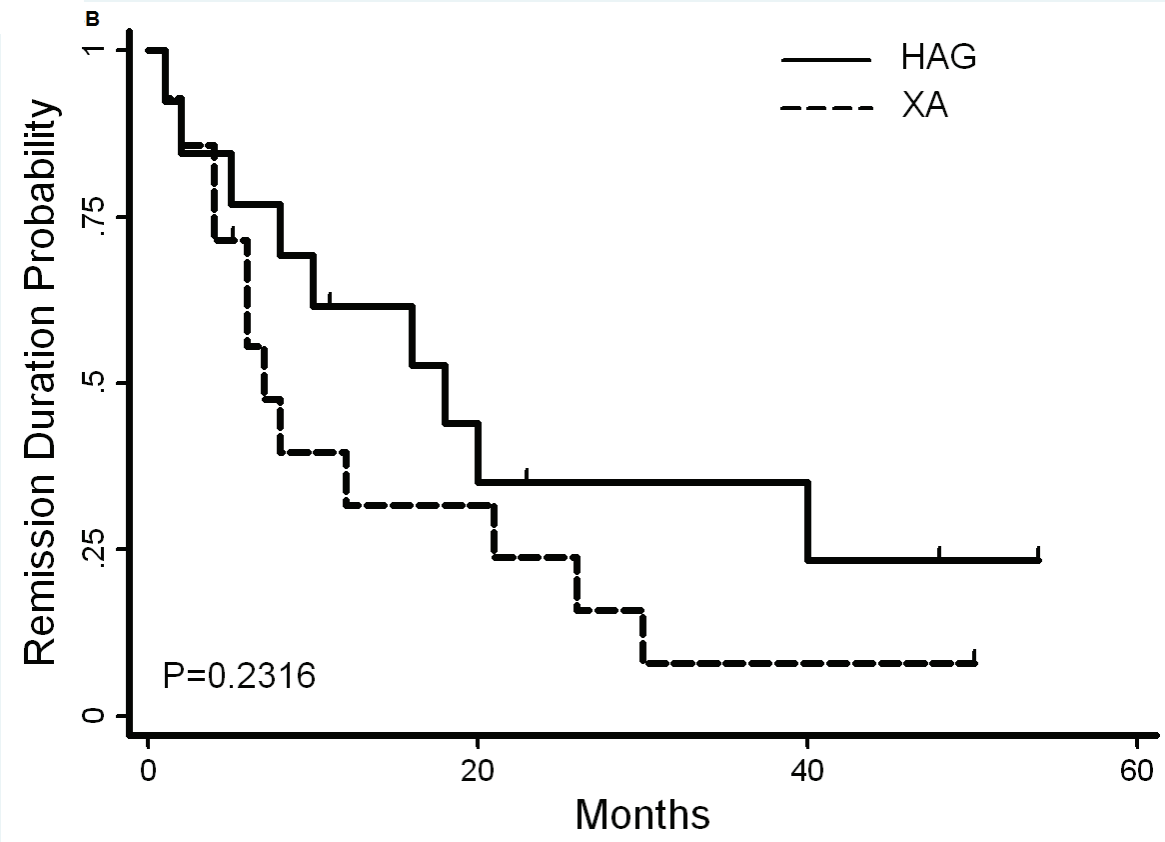
Overall survival and disease-free survival (DFS) of AML patients with hypocellular characteristics induced with XA or HAG The median survival time for patients induced with XA and HAG was 7 and 16 months, respectively **A.,** *P* = 0.02. The median disease-free survival (DFS) was 6.5 and 16 months, respectively **B.,** *P* = 0.2

The median follow up time was 12 months. For patients with CR, the median OS in the HAG and XA groups was 16 months and 7 months, respectively (*P* = 0.02, Table 3, Figure 3A), while the median DFS was 16 months and 6.5 months, respectively (*P* = 0.23, Table 3, Figure 3B).

Six patients younger than 60-year old underwent allogeneic stem cell transplantation on achieving the first CR, and additional 3 patients underwent transplantation during CR2. Of the 9 patients who underwent allogeneic transplantation, 4 patients belonged to the HAG group, of whom 3 patients remain alive; 5 patients belonged to the XA group, of whom 3 patients remain alive at the time of writing this report. Most CR patients (24/27) recovered to normal hematopoiesis; among the patients who relapsed, hypocellular hematopoiesis recurred in 12 patients, while 3 patients developed hypercellular AML.

The CR rate for hypocellular and non-hypocellular AML was 62.8% and 56.0%, respectively (*P* = 0.48). Twenty-seven (62.8%) patients with hypocellular AML were treated with XA regimen and their CR rate was 51.9%, with a median OS and DFS of 7 months and 6.5 months, respectively. Sixteen (37.2%) patients were treated with HAG and the CR rate in this cohort was 81.25% with a median OS and DFS of 16 months each. (Statistically better than the cohort treated with XA, Figure 2).

### Toxicity Profile of HAG regimen

During remission induction, 5 out of 27 patients (18.5%) in the XA group, and 6 out of 16 (37.5%) patients in the HAG group developed reversible grade I-II liver dysfunction.(*P* = 0.365). Normal hepatic function was restored in all of these patients after termination of induction therapy. Abnormal electrocardiogram (ECG.) findings were observed in 6 of 27 patients in the XA group, and 5 of 16 patients in the HAG group (*P* = 0.471). On further investigation, however, the ECG alteration was not deemed to be of any pathophysiological significance. In general, the results of laboratory study and examination were comparable between the two groups. (Table S1).

## DISCUSSION

In this study, we examined the incidence of hypo-AML and undertook a systematic analysis of the clinical and laboratory characteristics in AML patients. The incidence of hypo-AML in our study was 7.4% of all AML, which is similar to that in the previous report (Table 6). [9] However, it is remarkable that the frequency of hypo-AML was also comparable to that reported earlier (5-7%) using the criteria of bone marrow cellularity of 40-50% for labeling a case as that of hypo-AML. The frequency was expected to be lower than that computed using the criteria of < 20% bone marrow cellularity. The similar frequency calculated with different diagnostic criteria could possibly be attributable to the differences in the diagnostic criteria for AML itself. Earlier, the diagnostic criteria for AML required at least 30% myeloblasts in the bone marrow, which would decrease the incidence of AML, as the patients with 20-30% myeloblasts would then be diagnosed as cases of MDS-RAEB-T. In our study, the patients with 20-30% myeloblasts in bone marrow were diagnosed as AML, which is probably responsible for the increased yield of hypo-AML patients.

**Table 6:** Summary of reports on hypocellular acute myeloid leukemia

	Our study	Al-Kali [9]	Naseem S [10]	Nagai K [6]
**Reference time period**	2003-2013	2000-2009	2012-2013	Before 1992
**No. of patients, hAML/all AML**	43/541	123/1342	8/316	32
**Age, y (range)**	56 (20-65)	65 (19,88)	44 (16-50)	67 (44,75)
**Female, %**	21 (48.8)	NA	3 (37.5)	9 (28.1)
**AHD, N (%)**	10 (23.3)	41 (33.3)	4 (50)	N.A.
**WBC count, ×10^9^/L (range)**	2.2 (0.2-64.4)	1.7 (0.2,64.4)	2.65 (1.5,4)	1.45 (0.55, 4.5)
**HGB level, g/L (range)**	83 (30-142)	87 (30,142)	76 (48,111)	81 (38, 132)
**PLT count, ×10^9^/L (range)**	51 (4-306)	57 (4,306)	42.5 (5,391)	52.5 (1.5, 262.5)
**BM cellularity, % (range)**	14 (11-20)	15 (4-20)	≤ 40%	29.8 (12.4-39.8)
**Induction regimen**	“XA” HAG	IDA Clofarabine ± AraC	NA	LD-AraC
**CR rate, %**	62.8	53.7	NA	65
**Overall survival**	9 months	38 weeks	NA	NA
**Event/disease-free Survival**	8 months	13 weeks	NA	NA

hAML, Hypocellular acute myeloid leukemia; AHD, anterior hematological disease; CR, complete remission

The median age of subjects in our study (56 years) was lower than that in the MD Anderson's report (65 years). In our study, 10 patients had ADH (6 MDS and 4 MPN), which is different from the report from the MD Anderson group that considered a wider spectrum of disease including MDS, non-Hodgkin lymphoma (NHL), Hodgkin lymphoma (HL) and other non-hematopoietic neoplasms with a history of chemotherapy (Table 6). The difference in the disease spectrum might be due to lower number of patients with ADH in our study. Nonetheless, the other clinical characteristics of hypo-AML patients were similar to those reported earlier. The outcomes of hypo-AML did not appear to differ from that of non-hypocellular AML. In our study, the OS and DFS were comparable between hypo-AML and non-hypocellular AML patients. (Figure 2)

A notable finding from our study was a higher CR in hypo-AML patients treated with HAG regimen, as compared to those treated with XA, and that the increased CR rate did translate into prolonged OS and DFS. (Figure 3) On univariate analysis of induction failure in our study, AHD and poor cytogenetics markedly correlated with induction failure. On multivariate analysis, induction treatment regimen was observed to be an independent adverse prognostic factor for induction success along with other factors such as cytogenetics. Retrospectively, induction with HAG regimen is expected to benefit the adult AML patients with hypocellular bone marrow conferring an increased likelihood of achieving CR. The early death rate during HAG induction (6.25%, 1/16) was lower than that associated with XA induction (14.8%, 4/27) and cytarabine based induction (14%, 17/123) employed in MD Anderson's report.[9]

Consistent with results from earlier studies, HAG regimen is highly effective in refractory or relapsed AML patients, with the reported CR rates varying from 43% to 70%.[11-13] Similarly, another study showed that fifty percent (18/36) refractory and/or relapsed AML patients achieved CR with a median CR duration of 7.2 months.[11] These studies suggest HAG regimen to be highly effective in the treatment of refractory or relapsed AML. In the present study, patients with older age, AHD, and poor performance status tended to receive HAG induction, but they still achieved better survival rates as compared to that in the XA group.

The combination of cytarabine and anthracycline remains the cornerstone of chemotherapy for AML patients, with an associated remission rate of 70-75% in patients with *de novo* disease. In our study, XA regimen induced CR in 51.9% of patients. In MD Anderson's report, high dose cytarabine based regimens were used for two-thirds of patients, and induced 54% of patients with CR, while the early death rate was high at 14%. These results suggest that intensive chemotherapy induction might not be suitable for hypo-AML. The HAG regimen did show the advantage in the therapy of hypo-AML. In HAG group, the CR rate was 81.25%, with a prolonged OS and DFS. Less intensive regimens like HAG appear to be optimal in hypo-AML patients. The higher sensitivity of hypo-AMLs to low dose cytarabine or melphalan has been documented. With low-dose cytarabine regimen, a significantly higher CR rate (65%) was achieved in hypo-AML than in RAEB/RAEB-t (0%) and overt AML in the elderly cases (27.3%).[6] Low-dose melphalan (oral 2 mg/d) has been reported as being highly effective in inducing complete remission in relatively high-risk patients such as, elderly patients with AML, AML with multilineage dysplasia and those with MDS.[14,15,16] The HAG regimen contains low-dose cytarabine and G-CSF, which may contribute to the higher CR rate observed in patients with hypo-AML. Others have also described effective induction of remission with G-CSF [17, 18], possibly by inducing differentiation of leukemic blasts in the hypo-AML.[19] G-CSF is known to induce the differentiation of leukemic cells into the monocytic lineage, which causes apoptosis of leukemic cells, both *in vivo* and *in vitro*.[20] G-CSF stimulates the leukemia initiating cells into cell cycle, induces more leukemia cells sensitive to cytarabine, and enhances normal hematopoiesis.[19] CAG regimen which is similar to HAG, comprises of cytarabine, aclarubicin and G-CSF, which has been widely used in China and Japan for treatment of AML and MDS. A meta-analysis showed a significantly higher CR rates associated with CAG as compared to those of non-CAG regimens (odds ratio 2.43). [21] These findings are consistent with our observations in the present study. The small sample size is one of the limitations of our study which precludes any definitive conclusions to be drawn. Our results just suggest that lower intensive regimen like HAG might be the optimal induction regimens for hypo-AML. Large, multi-center prospective studies are required to attain a comprehensive understanding of this disease entity.

In summary, the rare occurrence of hypo-AML is a challenge to its in proper diagnosis and rational treatment. In this study, use of HAG regimen as induction therapy was associated with superior outcomes and was an independent prognostic factor for OS.

## DESIGN AND METHODS

### Patients' details

Adult patients with newly diagnosed AML at the Department of Hematology, Changhai and Changzheng Hospital between January 1^st^, 2003 and December 31^st^, 2013 were retrospectively analyzed, excluding acute promyelocytic leukemia (APL) during the same period. The study protocol was approved by the Ethics Committee at the Second Military Medical University. Written informed consent was obtained from all patients (or their legal guardians) for treatment and prospective data collection in accordance with the Declaration of Helsinki. AML diagnosis was typed and characterized according to the WHO and FAB classification schemes. [22, 23]

### Bone marrow aspirate and pathology

According to the clinical practice at our institute, bone marrow aspirate smears with core biopsies were performed on every patient at the time of presentation. Bone marrow aspirate smears were assessed by Wright-Giemsa stain followed by their cytochemical analysis.

All cases with hypo-AML were reviewed by an independent pathologist including examination of peripheral blood smears, bone marrow aspirate smear and the bone marrow biopsies. The diagnosis criteria for hypo-AML adopted by the French-American-British Cooperative Leukemia Working Group in 2009 were used. [5] A bone marrow or blood blast count ≥ 20% in ANCs is required for a diagnosis of AML. If erythroid precursors in bone marrow account for ≥ 50% of bone marrow ANCs, then myeloblast count of ≥ 20% in non-erythroid cell population is required. A concomitant finding of bone marrow biopsy cellularity of < 20% is obligatory.

### Immunophenotyping analysis

Seven-color flow cytometry immunophenotypic analysis was performed on bone marrow aspirate specimens using a fluorescence-activated cell sorting (FACS) FACSAria II, as described previously.

### Cytogenetics analysis

Direct and short-term culture methods were used for preparation of bone marrow specimens. Chromosome banding was carried out by heating using the R-banding method, with an average of 20 metaphase cells analyzed in each case. Karyotype was determined according to the International System for Human Cytogenetic Nomenclature (ISCN, 1995). Cytogenetic data was stratified into three subgroups (modified from previous complex karyotype [24-26]): Poor-risk (−5,-7, +8 and 11q involvement and complex karyotype), good-risk (t (8; 21) or Inv (16) or t (16; 16)) and intermediate-risk (neither good nor poor).

### Mutational analysis

Bone marrow was the source of DNA in all cases. We sequenced the entire coding regions of NPM1 and FLT3-ITD. Paired remission DNA (i.e., DNA from patients who had a complete remission after induction chemotherapy) was available from 37 of the 43 participants. Data on variants that could not be validated as bona fide somatic mutations due to unavailable remission DNA and the absence of reports, the mutations in the published literature of somatic mutational status for that specific gene was used.

### Treatment

Two kinds of induction chemotherapy regimen were used in our protocol for Hypo-AML. One was the standard regimen (“XA” regimen), which is comprised of daunomycin 45∼60 mg/m^2^/d 1-3 or idarubicin 8∼10 mg/m^2^/d 1-3, or mitoxantrone 8 mg/m^2^/d 1-3 plus with cytarabine 100∼150 mg/m^2^/d 1-7. The other was HAG regimen, which is comprised of homoharringtonine (HHT, 1 mg/day, intravenously, on days 1-14); cytarabine 25 mg/m^2^/24h divided into twice, subcutaneously, on days 1-14, and G-CSF 5 μg/kg/d from d0 until the neutrophil counts above 1.5×10^9^/L. In XA cohort, G-CSF was given to patients at 48 hours after the end of chemotherapy while the neutrocyte was lower than 1.5×10^9^/L. For patients with WBC counts above 30×10^9^/L, hydroxyurea was given to decrease the WBC counts to less than 10×10^9^/L for initiating chemotherapy with HAG regimen. The G-CSF priming was discontinued when WBC counts reached above 30×10^9^/L. Up to two cycles of induction therapy were allowed if CR was not achieved. Patients who achieved PR with one cycle of induction were re-induced with the same regimen.

Patients with CR were consolidated with four cycles of intermediate-dose cytarabine (ID-Ara-C, 2 g/m^2^, q12h, for 6 doses). 9 patients received allogeneic hematopoietic stem cell transplantation with eligible matched related or unrelated donors after the attainment of CR. The conditioning regimen included busulfan (3.2 mg/kg/d × 4 days) and cyclophosphamide (60 mg/kg/d × 2 days).

### Criteria for response and definition of disease-free survival

CR was defined by the presence of < 5% blasts in the bone marrow, absence of extramedullary leukemia, and peripheral blood count recovery with a neutrophil count of at least 1×10^9^/L and platelet count of at least 100×10^9^/L.[27, 28] PR was defined as 5-15% blasts with a decrease of at least 50% reduction of bone marrow blasts at diagnosis. Relapse was defined by an excess of 5% leukemic blasts in a marrow aspirate unrelated to recovery of normal hematopoiesis, or the development of new extramedullary leukemia. DFS was calculated from the date of first CR. All patients were followed up from the time of their diagnosis until the end of December, 2014.

### Statistical methods

Chi-squared tests and non-parametric Mann-Whitney tests were used for descriptive statistical analysis of categorical or continuous variables. The impact of pathological feature on OS and DFS was estimated using the Kaplan-Meier method and compared using log rank analysis.[29] For multivariate analysis, stepwise Cox proportional hazard (95% confidence intervals [CI]) were performed using R 2.7.2 software package.

## SUPPLEMENTARY MATERIAL TABLE


