# Supplementary material for: HAG regimen improves survival in adult patients with hypocellular acute myeloid leukemia
Source: Oncotarget. 2015 Oct 21;7(3):3623–34. doi: 10.18632/oncotarget.6211 (PMC4823132; doi:10.18632/oncotarget.6211)
Supplement: Supplementary file 1 [file oncotarget-07-3623-s001.pdf]

HAG regimen improves survival in adult patients with hypocellular acute myeloid leukemia

Supplementary Material

Table S1. Cardiac and liver toxicity during XA and HAG induction.

	XA	HAG	*P*
**No. of patients**	27	16	
**Abnormal E.C.G.**			0.471
T wave changes	4 (14.8%)	2 (12.5%)	
RBBB	1 (3.7%)	2 (12.5%)	
LBBB	1 (3.7%)	0	
Long QT interval	0	1 (6.25%)	
**Abnormal liver function**			0.365
ALT	3 (11.1%)	3 (18.7%)	
AST	2 (7.4%)	3 (18.7%)	

RBBB, Right bundle branch block; LBBB, Left bundle branch block; ALT, alanine aminotransferase; AST, aspartate aminotransferase
